# A transcriptional evaluation of the melanoma and squamous cell carcinoma TIL compartment reveals an unexpected spectrum of exhausted and functional T cells

**DOI:** 10.3389/fonc.2023.1200387

**Published:** 2023-10-30

**Authors:** Cheryl M. Cameron, Brian Richardson, Jackelyn B. Golden, Yee Peng Phoon, Banumathi Tamilselvan, Lukas Pfannenstiel, Samjhana Thapaliya, Gustavo Roversi, Xing-Huang Gao, Leah L. Zagore, Mark J. Cameron, Brian R. Gastman

**Affiliations:** ^1^ Department of Nutrition, Case Western Reserve University, Cleveland, OH, United States; ^2^ Department of Population and Quantitative Health Sciences, Case Western Reserve University, Cleveland, OH, United States; ^3^ Department of Inflammation and Immunity, Cleveland Clinic, Cleveland, OH, United States; ^4^ Department of Plastic Surgery, Cleveland Clinic, Cleveland, OH, United States

**Keywords:** melanoma, T cells, tumor-infiltrating cells, immune checkpoint receptor, RNAseq

## Abstract

**Introduction:**

Significant heterogeneity exists within the tumor-infiltrating CD8 T cell population, and exhausted T cells harbor a subpopulation that may be replicating and may retain signatures of activation, with potential functional consequences in tumor progression. Dysfunctional immunity in the tumor microenvironment is associated with poor cancer outcomes, making exploration of these exhausted T cell subpopulations critical to the improvement of therapeutic approaches.

**Methods:**

To investigate mechanisms associated with terminally exhausted T cells, we sorted and performed transcriptional profiling of CD8^+^ tumor-infiltrating lymphocytes (TILs) co-expressing the exhaustion markers PD-1 and TIM-3 from large-volume melanoma tumors. We additionally performed immunologic phenotyping and functional validation, including at the single-cell level, to identify potential mechanisms that underlie their dysfunctional phenotype.

**Results:**

We identified novel dysregulated pathways in CD8^+^PD-1^+^TIM-3^+^ cells that have not been well studied in TILs; these include bile acid and peroxisome pathway-related metabolism and mammalian target of rapamycin (mTOR) signaling pathways, which are highly correlated with immune checkpoint receptor expression.

**Discussion:**

Based on bioinformatic integration of immunophenotypic data and network analysis, we propose unexpected targets for therapies to rescue the immune response to tumors in melanoma.

## Introduction

Immune checkpoint inhibition (ICI) has revolutionized the treatment of many forms of cancer, especially UV-induced skin cancers like melanoma (1–6). The success of ICI has also escalated the need for a comprehensive mechanistic understanding of the role that cellular ICI targets play in oncogenesis and response to treatment. Furthermore, this successful clinical outcome has prompted a search for additional checkpoints and negative regulators of T cell function that can lead to new treatment options for the substantial subset of patients who are refractory (1–3, 5–7).

Systems biology has revolutionized the comprehensive objective assessment of the state of T cells in myriad disease models. Transcriptional analysis via RNASeq has transformed the field of tumor immunology and facilitated the identification of new subpopulations within what was previously thought to be a homogeneous set of immune populations (8, 9). The vast majority of the literature on melanoma, however, relies on small biopsies, so it is likely that the data may not represent the full spectrum of alterations occurring in the immune system in the tumor microenvironment (TME) (10, 11). An integrated systems biology approach may yield key transcriptomic biological maps of the TME (and/or identify gaps) to provide insight into the delicate balance of regulation within the tumor and in immune cells to pinpoint therapeutically targetable states within the spectrum of function. We have a unique biobank of fresh, large-volume tumors from surgical resections, allowing us to sort down to very specific individual populations to investigate subset-specific transcriptional regulation for discovery.

Although many immune cells in the TME express immune checkpoints, such as programmed cell death-1 (PD-1), it is the exhausted tumor-infiltrating CD8^+^ T cell that has been shown to be a key target for reinvigoration by ICI (12–17). T cell exhaustion is a dynamic process, associated with progressive loss of effector function and high expression of inhibitory receptor molecules, such as PD-1, LAG-3, and TIM-3 (18) (19). Interestingly, in the last few years, several transcriptomic evaluations of CD8+ TILs have identified exhausted T cell populations with high levels of activation (20) and even significant populations that are proliferating (21), contrasting with reports that these exhausted cells are dysfunctional (22, 23). Few studies have investigated these TILs, even though they express the same targets of modern ICI and the efficacy of immune checkpoint blockade therapy is significantly influenced by exhausted T cell heterogeneity (24). Recent studies have shown that T cells can be separated into defined phenotypic and functional categories. Current models posit that T cells exposed to chronic high levels of antigen, as in unresolved infection and tumor, contain populations of “precursor exhausted” cells (Tpex: PD1^+^, TCF1- and TIM-3-) and “terminally exhausted” T cells (Tex: PD-1^+^, TCF1-, and TIM-3^+^) (25). While both subpopulations express PD-1, a canonical marker of T cell exhaustion, Tpex cells have been shown to display characteristics of both exhausted and memory cells and proliferate in response to blocking inhibitory receptors, differentiating into Tex cells (26) (27) (28). We recently showed that cells with a Tex phenotype (CD8^+^PD-1^+^TIM-3^+^ TILs) functionally mediate suppression of healthy autologous T cell proliferation through the production of IL10 and close contact. Importantly, these cells are a target of modern checkpoint-based immunotherapy (29, 30). These studies highlight the need to investigate all exhausted T cell populations. Further research into the dynamic regulation of and functional interplay between exhausted T cell subsets and the TME may serve to facilitate future therapeutic approaches.

We hypothesized that investigation of CD8^+^PD-1^+^TIM-3^+^ T cells (Tex/act, exhausted but actively suppressive) would yield key transcriptomic information that can be exploited to identify new targets for immunotherapy. We strategically sorted and transcriptomically profiled CD8^+^PD-1^+^TIM-3^+^ TILs from melanoma and from cutaneous squamous cell carcinoma (SCC) tumors to answer these questions. We undertook what appears to be the largest unbiased transcriptomic evaluation (in terms of the number of patients) of this key cellular ICI target within the TME and identified numerous differentially regulated biochemical pathways. Of note, these CD8^+^PD-1^+^TIM-3^+^ T cells are metabolically active TILs and demonstrate profound upregulation of pathways including bile acid metabolism, mammalian target of rapamycin (mTOR) signaling, and peroxisome-related pathways. Given the fact that there is still a large group of melanoma and other cancer patients who do not derive benefit from ICI or other therapies, our findings may have broad application in helping to reach the goal of therapeutic success in all patients.

## Results

### Transcriptomic profiling of dysfunctional CD8^+^ TILs

**Figure 1:**
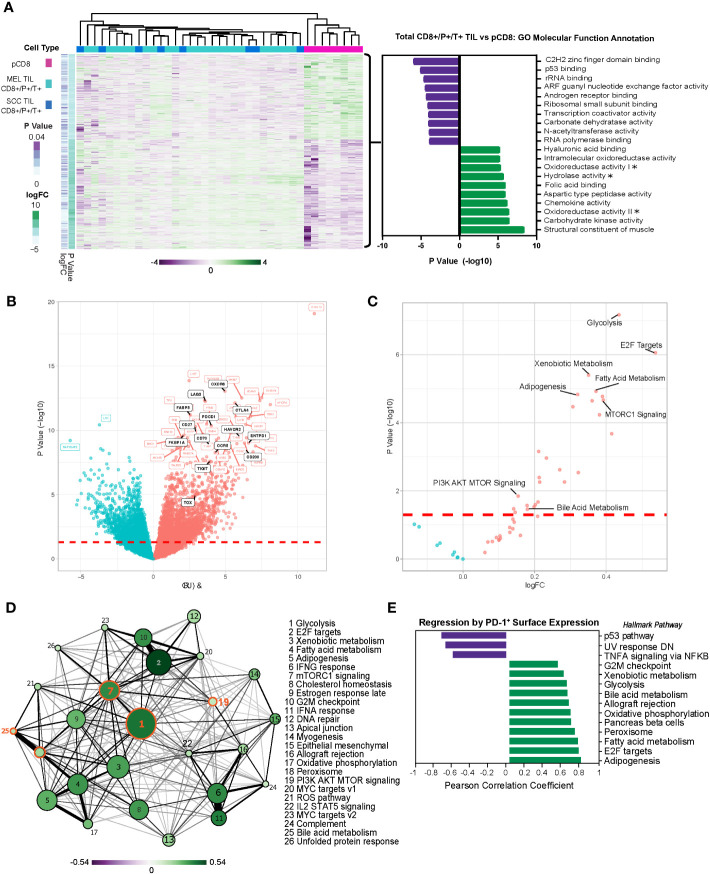
Melanoma and SCC TILs display a distinct transcriptional profile from CD8^+^ T cells in the periphery. **(A)** Heatmap of 3,957 differentially expressed genes (DEGs) (nominal p < 0.05) in PD-1^+^ TIM-3^+^ TILs from melanoma (MEL TILs CD8^+^/P^+^/T^+^) and SCC (SCC TILs CD8^+^/P^+^/T^+^) *vs.* pCD8 cells. Gene expression is normalized by z-score, with green indicating higher relative levels of expression and purple indicating lower relative levels of gene expression. GO molecular function analysis (*via* GSVA) of DEGs in melanoma and SCC CD8^+^/P^+^/T^+^ TILs (total CD8^+^/P^+^/T^+^ TILs) *vs.* pCD8s is shown to the right of the heatmap. Asterisks indicate truncated pathway names; full pathways are as follows: oxidoreductase activity acting on the CH–NH group of donors (oxidoreductase activity I), oxidoreductase activity acting on the CH–NH2 group of donors with oxygen as acceptor (oxidoreductase activity II), and hydrolase activity acting on carbon–nitrogen but not peptide bonds in cyclic amidines (hydrolase activity). **(B)** Volcano plot of the top 50 up- or downregulated genes in total CD8^+^/P^+^/T^+^ TILs *vs.* pCD8s. Upregulated genes are shown in red, downregulated genes are shown in blue, and significant genes of interest to this study are annotated in black. The red dashed line indicates the significance cutoff (nominal p < 0.05). **(C)** Volcano plot of Hallmark pathway enrichment in total CD8^+^/P^+^/T^+^ TILs *vs.* pCD8s. Significant pathways of interest are annotated; the red dashed line indicates the significance cutoff (nominal p < 0.05). **(D)** Enrichment map of significantly differentially enriched Hallmark pathways (nominal p < 0.05) in total CD8^+^/P^+^/T^+^ TILs *vs.* pCD8s. Node size indicates negative (−)log10(p-value), node color with associated scale denotes log2 fold change values for enrichment scores, and edge weight represents the Jaccard coefficient between significant sets of genes in each pathway. **(E)** Regression of PD-1 (GEOMEAN) surface expression on CD8^+^ T cells on sample pathway enrichment scores from the Hallmark database. Pathway enrichment scores are normalized by z-score, with green indicating higher relative levels of enrichment and purple indicating lower relative levels of enrichment. SCC, squamous cell carcinoma; TILs, tumor-infiltrating lymphocytes; GO, gene ontology; GSVA, gene set variation analysis.

Melanoma and SCC have shared etiologies (e.g., both are UV-induced cutaneous conditions) and similar immune-oncology-based therapeutic approaches are adopted in treating them, but these conditions can progress very differently, lending themselves to the interrogation of CD8 T cell exhaustion and analysis for common or distinct mechanisms of dysfunction with applications for the development of improved therapies. CD8^+^ TILs in melanoma and SCC share important common features, including the coordinated expression of immune checkpoint receptors and the development of resistance to immunotherapy (29, 31, 32), yet the mechanisms underlying TIL dysfunction in these tumors remain elusive. To address this, we performed transcriptomic profiling on flow-sorted CD8^+^PD-1^+^TIM-3^+^ T cells from melanoma and SCC (total CD8^+^/P^+^/T^+^ TILs) and compared them to peripheral CD8^+^ T cells (pCD8s) from healthy donors. A total of 3,957 genes were differentially expressed (p ≤ 0.05) in total CD8^+^/P^+^/T^+^ TILs *vs.* pCD8^+^ T cells (**Figure 1A**). Gene set variation analysis (GSVA), beginning with gene ontology (GO) molecular function annotation, identified the corresponding top upregulated pathways, including chemokine activity and three pathways related to metabolism: carbohydrate kinase activity, oxidoreductase activity II, and aspartic-type peptidase activity (**Figure 1A**). The top downregulated pathways included C2H2 zinc finger domain binding, p53 binding, rRNA binding, ARF guanyl nucleotide exchange factory activity, and androgen receptor binding (**Figure 1A**). Clear differential expression of genes in total CD8^+^/P^+^/T^+^ (SCC and MEL) TILs *vs.* pCD8s indicated upregulation of immune checkpoint receptors including TIM-3 (HAVCR2), PD-1 (PDCD1), LAG3, and TIGIT (**Figure 1B**).

Moreover, additional pathway enrichment analysis via gene set enrichment analysis (GSEA) using the Hallmark database (MSigDB) identified significant induction of multiple metabolic pathways in total CD8^+^/P^+^/T^+^ TILs, including glycolysis, xenobiotic metabolism, fatty acid metabolism, mTORC1 signaling/PI3K AKT MTOR signaling, adipogenesis, and bile acid metabolism, corroborating our GSVA data (**Figure 1C**). We performed an analysis of pathway interactivity (*via* shared differentially expressed genes (DEGs) according to Jaccard similarity coefficient) to provide a broader overview of how the enriched pathways interact; as expected, this indicated a close correlation between mTORC1 signaling, PI3K AKT MTOR signaling, and glycolysis, with a less-direct connection to bile acid metabolism (*via* mTORC1 signaling) (**Figure 1D**). We were especially intrigued by the significant differential enrichment of bile acid metabolism, which has not been proposed as a key regulatory pathway in TIL function. Linear regression analysis of PD-1 (surface expression determined by flow cytometry) with gene expression revealed a strong positive correlation between PD-1 protein expression and upregulation of the bile acid metabolism, peroxisome, and glycolysis pathways in CD8^+^ TILs and peripheral blood mononuclear cells (PBMCs) (**Figure 1E**). These findings suggest that CD8^+^ T cells adopt a unique metabolic program associated with the latest stages of exhaustion when PD-1 expression is at its highest.

Moreover, core biological pathways that have been previously shown to be associated with TGF-β attenuation of the tumor response to immune checkpoint blockade were also significantly differentially regulated within the dysfunctional CD8^+^PD-1^+^TIM-3^+^ melanoma and SCC TILs (**Supplementary Figure 1A**).

### Identification of unique metabolic-associated dysfunctional CD8^+^ T cell clusters by UMAP analysis

**Figure 2:**
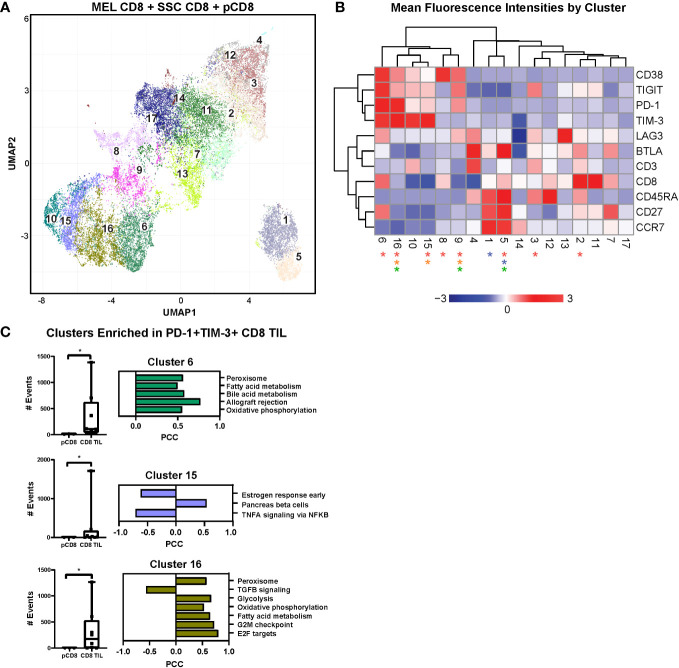
UMAP analysis and linear regression with immune checkpoint cell surface markers. **(A)** UMAP dimensional reduction analysis of CD8^+^ TILs compared to CD8^+^ PBMCs reveals 17 unique clusters of coordinate cell surface protein expression. **(B)** Two-way hierarchically clustered heatmap of the MFI (geometric mean fluorescence intensity) of surface expression of CD38, TIGIT, PD-1, TIM-3, LAG3, BTLA, CD3, CD8, CD45RA, CD27, and CCR7 within the clusters in the UMAP analysis. Clusters significant for all TILs *vs.* pCD8s are indicated by red asterisks (2, 3, 5, 6, 8, 9, 15, and 16), for MEL TILs *vs.* SCC TILs by blue asterisks (1 and 5), for MEL TILs *vs.* pCD8s by orange asterisks (9, 15, and 16), and for SCC TILs *vs.* pCD8s by green asterisks (5, 9, and 16). Heatmap is z-score transformed with upregulated MFI in red and downregulated MFI in blue. **(C)** Individual counts per selected cluster are shown in the box-and-whisker plots (clusters 6, 15, and 16 are notably enriched in all TILs compared to pCD8 cells; p < 0.05, Mann–Whitney test). Linear regression modeling of each cluster with the RNAseq data reveals enriched pathways that are unique to each cluster. UMAP, uniform manifold approximation and projection; TILs, tumor-infiltrating lymphocytes; PBMCs, peripheral blood mononuclear cells; MEL, melanoma; SCC, squamous cell carcinoma.

To objectively investigate additional exhausted T cell phenotypes and associated metabolic signatures, we integrated the expression of immune checkpoint receptors (ICRs) and activation markers with the transcriptional profiles of CD8^+^ TILs (melanoma and SCC) and peripheral CD8^+^ T cells (**Figure 2**). We used flow cytometric analysis of a smaller, randomly selected subset of samples to measure the expression of immune checkpoint receptors PD-1, TIM-3, TIGIT, BTLA, LAG3, and CD38, which have all been shown to regulate T cell function (33). Coordinate expression of multiple ICRs is associated with progressive CD8^+^ TIL dysfunction; therefore, we used our flow cytometry data to infer levels of CD8^+^ TIL exhaustion.

We employed uniform manifold approximation and projection (UMAP), a non-linear dimensionality-reduction machine learning approach, to analyze our multiparametric flow data and objectively determine the heterogeneity of CD8^+^ T cells based on surface expression of BTLA, CD3, CD8, CD27, CD38, CD45RA, CCR7, LAG3, PD-1, TIGIT, and TIM-3. UMAP analysis revealed 17 unique clusters, nine of which showed significantly different frequencies in CD8^+^ TILs compared to PBMCs (**Figure 2A**). A two-way hierarchically clustered heatmap of MFI (geometric mean fluorescence intensity) based on surface expression shows the distinct surface signature profiles of the 17 clusters identified by UMAP analysis (**Figure 2B**). Notably, the frequencies of lymphocytes co-expressing multiple immune checkpoints (CD38, TIGIT, LAG3, PD-1, and TIM-3), the negative regulators of T cell function, were enriched in CD8^+^ TILs compared to peripheral cells, especially in clusters 6, 15, and 16.

Of note, cluster 6 represents what is likely to be the most highly dysfunctional CD8 population, characterized by relative upregulation of CD38, TIGIT, PD-1, LAG3, and TIM-3, and is significantly enriched in the CD8^+^ TIL population compared to the peripheral CD8^+^ T cells (**Figures 2B, C**). Similar to cluster 6, clusters 10, 15, and 16 have similar ICR expression patterns with increased coordinate immune checkpoint expression levels (**Figure 2B**). Interestingly, cluster 16 is significantly enriched in CD8^+^ TILs of both melanoma and SCC with high expression of PD-1 and TIM-3 (**Figures 2B, C**). Likewise, cluster 15 is also enriched in CD8^+^ TILs, but only in melanoma, with moderate immune checkpoint expression (**Figures 2B, C**). Our findings indicate that clusters 6, 10, 15, and 16, with upregulation of ICRs, are likely to represent progressively more exhausted CD8^+^ T cell clusters (**Figure 2B**).

We then performed linear regression modeling of gene expression with frequencies of cells from each flow UMAP cluster in each donor to identify transcriptomic pathway enrichment that correlates significantly with frequencies of CD8^+^ T cell clusters identified by UMAP. Interestingly, higher frequencies of cells in cluster 6 were strongly correlated with positive enrichment of the bile acid metabolism and peroxisome pathways (**Figure 2C**). Likewise, cluster 16 frequency was also positively correlated with enrichment of the peroxisome, oxidative phosphorylation, and glycolysis pathways (**Figure 2C**). In summary, our data demonstrate a significant correlation between the upregulation of immune checkpoint-expressing dysfunctional CD8^+^ TILs and activation of metabolic functions, particularly the bile acid metabolism, peroxidase, and glycolysis pathways, at the transcriptomic level. Targeting this metabolic-associated dysfunctional CD8^+^ Tex/act cell population could reinvigorate exhausted CD8^+^ T cells, thus enhancing immunotherapy responses in cancer patients.

### Upregulation of proliferation- and metabolic-associated genes in dysfunctional CD8^+^ TILs in melanoma

**Figure 3:**
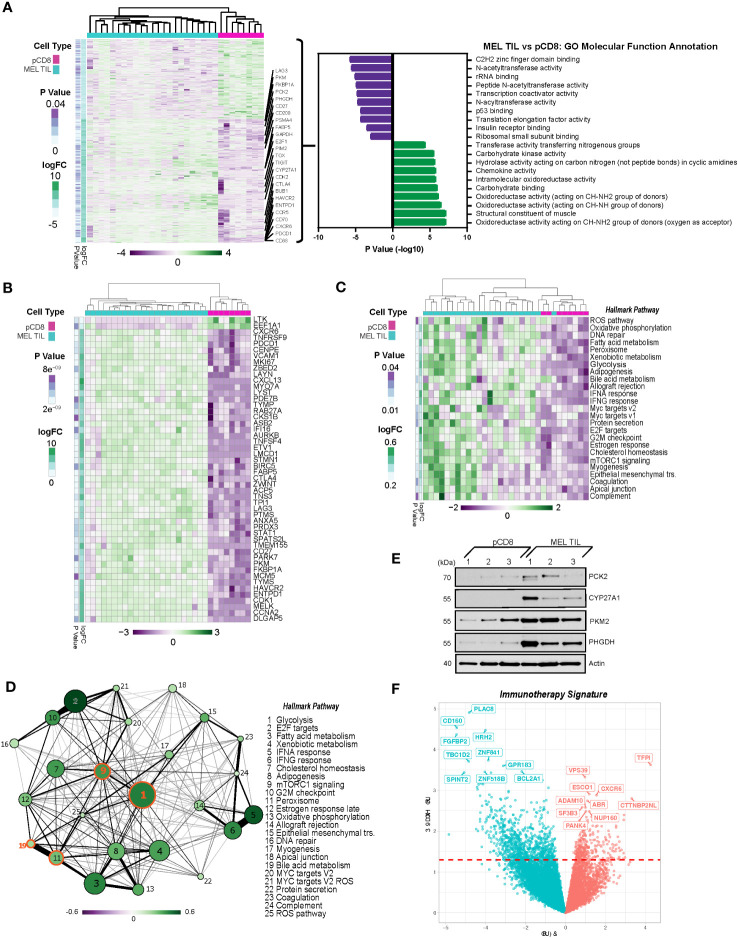
PD-1^+^TIM-3^+^ TILs from melanoma display a distinct transcriptional profile from CD8^+^ T cells in the periphery. **(A)** Heatmap of 4,280 differentially expressed genes (DEGs) (nominal p < 0.05) in TILs from melanoma *vs.* peripheral CD8 cells. Gene expression is normalized by z-score, with green indicating higher relative levels of expression and purple indicating lower relative levels of gene expression. Genes of interest are annotated. GO molecular function analysis (*via* GSVA) of DEGs in melanoma TILs *vs.* pCD8s is shown to the right of the heatmap. **(B)** Top 50 differentially expressed genes from MEL TILs *vs.* pCD8s based on p-value (nominal p < 8.93e−9). **(C)** Heatmap of significant (nominal p < 0.05) Hallmark pathways associated with pCD8s versus MEL TILs. **(D)** Enrichment map of significantly differentially enriched Hallmark pathways (nominal p < 0.05) in TILs from melanoma *vs.* pCD8s. Node size indicates −log10(p-value), node color with the associated scale denotes log2 fold change values for enrichment scores, and edge weight represents the Jaccard coefficient between the sets of significant genes in each pathway. **(E)** Western blot analysis of control pCD8^+^ T cells (n = 3) and melanoma TILs (n = 3). Protein targets are shown on the right y-axis; β-actin was used as a loading control. **(F)** Volcano plot of 1,072 differentially expressed genes (nominal p < 0.05) in patients who have undergone immunotherapy (anti-PD-1). Upregulated genes are shown in red, while downregulated genes are shown in blue; the top 10 up- and downregulated genes are annotated (red dashed line indicates p-value cutoff (nominal p < 0.05). TILs, tumor-infiltrating lymphocytes; GO, gene ontology; GSVA, gene set variation analysis; MEL, melanoma.

To determine whether these metabolic pathways were specifically dysregulated in melanoma, we performed differential gene expression analysis of the melanoma CD8^+^ TILs versus healthy peripheral CD8^+^ T cells, resulting in a distinct gene expression signature as shown by the heatmap in **Figure 3A**. A total of 4,280 genes were differentially expressed in MEL CD8^+^ TILs versus peripheral CD8^+^ T cells, while 2,057 of those genes were commonly differentially regulated in MEL and SCC TILs (p ≤ 0.05, **Supplementary Figure 1B**). Statistically, the most upregulated pathways based on GO molecular function annotation (*via* GSVA) were the oxidoreductase, structural constituent of muscle, and carbohydrate-binding pathways (**Figure 3A**). The top downregulated pathways included C2H2 zinc finger domain binding, *N*-acetyltransferase activity, rRNA binding, peptide *N*-acetyltransferase activity, and transcription coactivator activity. The top 50 differentially regulated genes between MEL TILs and pCD8s by statistical significance are shown in **Figure 3B**, and the ICRs PD-1 (PDCD1), TIM-3 (HAVCR2), CTLA4, and LAG3 were among these. Also significantly upregulated were FABP5 (epidermal fatty acid binding protein), which can act as an intracellular receptor that binds to free fatty acids (FFA) in the cytosol, and Ki-67 (MKI67), indicating enhanced proliferation of the CD8^+^ TILs.

### Enrichment of metabolism pathways in CD8^+^ TILs in melanoma

To further evaluate the biology underlying differentially expressed genes found in CD8^+^ TILs and PBMCs in melanoma, we performed pathway enrichment analysis of key canonical pathways. Notably, the peroxisome, bile acid, and fatty acid metabolism pathways were coordinately regulated in melanoma TILs compared to pCD8s (**Figure 3C**). Not only are peroxisome proliferator-activated receptor (PPAR) pathways associated with FABP5, which was upregulated at the RNA level in dysfunctional CD8^+^ TILs, but they also regulate bile acid synthesis (34–36). Our data showed strong positive enrichment of bile acid metabolism pathways in dysfunctional CD8^+^ T cells from melanoma tumors. CYP27A1, a key regulatory enzyme of the bile acid metabolism pathway, was significantly upregulated in the CD8^+^ T cells of melanoma TILs as compared to PBMCs (**Figure 3A**). Upregulation of additional genes from the bile acid and peroxisome signaling pathways was also evident in TIL gene and pathway expression analysis, including upregulation of SOD1, ACSL5, FADS2, CAT, DHCR24, SLC27A2, and IDH2 in melanoma TILs compared to pCD8s (**Supplementary Figure 2**).

As shown in **Figure 3C**, pathway enrichment analysis showed that the mTORC1 signaling pathway was significantly and positively enriched in CD8^+^ melanoma TILs as compared to pCD8s. The glycolysis pathway was highly integrated with many other pathways, particularly the mTORC1 and peroxisome pathways, which interact with bile acid metabolism (**Figure 3D**). Compared to pCD8 T cells from control individuals, the glycolysis pathway was the most significantly upregulated pathway under pathway interaction network analysis in the Hallmark canonical pathway database in CD8^+^ TILs from melanoma patients (**Figure 3D**). The differential regulation of multiple metabolic pathways, including bile acid metabolism, mTORC1, and glycolysis (**Figure 3D**), may reflect the fact that the CD8^+^ TIL cells are hosted in a hypoxic, acidic, and nutrient-depleted TME where the cellular metabolism has been reprogrammed as an adaptive mechanism to facilitate their proliferation and survival in melanoma. Our western blot analysis confirmed increased expression of CYP27A1, which was upregulated in the bile acid metabolic pathway, and three additional glycolytic genes (PKM2, PHGDH, and PCK2) in melanoma CD8^+^ TILs compared to PBMCs (**Figure 3E**).

### Identification of immunotherapy signatures

A subset of our cohort of melanoma patients were treated with anti-PD-1 immunotherapy. There were 1,072 differentially expressed genes (p ≤ 0.05) in patients receiving therapy (n = 5) as compared to those who did not receive treatment (n = 8), as summarized in the volcano plot in **Figure 3F**. CXCR6 was upregulated in the melanoma anti-PD-1 immunotherapy signature, indicating a potential shift toward a proinflammatory tumor microenvironment, which could lead to subsequent metastasis. CTTNBP2NL (CTTNBP2 N-terminal-like protein) was also upregulated; this is known to be associated with STRIPAK complexes, which have been broadly linked to metabolism, immune regulation, and cancer tumorigenesis (37). On the other hand, CD160 and HRH2 (histamine receptor H2) were downregulated in the anti-PD-1 therapy signature. To identify common features of immunotherapy treatment across multiple cancer types, we performed a similar analysis including patients with SCC and melanoma cancer types and multiple immunotherapy treatments. The comparison of patients receiving immunotherapy (n = 16) and those who did not (n = 14) revealed 1,118 differentially expressed genes (p ≤ 0.05). CYP27A1 and ABCA1 were among those genes significantly downregulated in patients receiving immunotherapy. Consistent with the anti-PD-1 therapy signature, we also saw downregulation of CD160 and HRH2 (**Supplementary Figure 3A**). Notably, pathways involved in fatty acid metabolism, mTORC1 signaling, glycolysis, and xenobiotic metabolism were all downregulated in the immunotherapy signature (**Supplementary Figure 3B**).

### mTOR pathway validation

**Figure 4:**
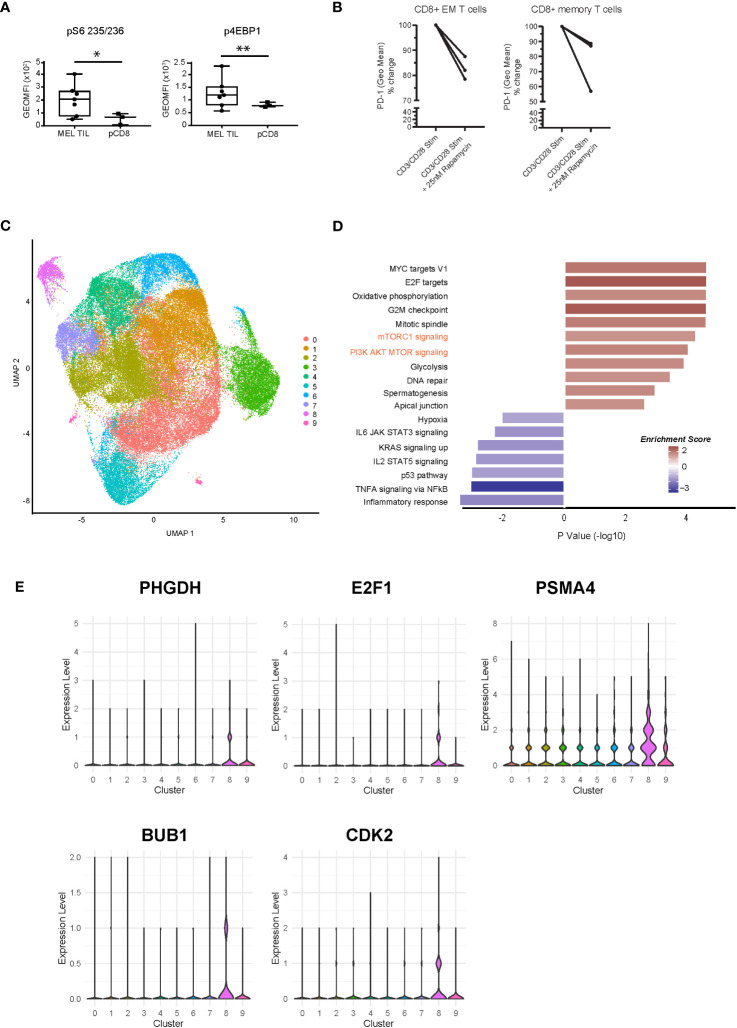
Dysregulation of the mTOR pathway at the phosphoprotein and single-cell transcriptomic level. **(A)** Intracellular flow cytometry of two phosphorylated targets, S6 ribosomal protein and 4EBP1, demonstrates significantly higher levels in CD8^+^ TILs of melanoma compared to peripheral CD8^+^ T cells. *p = 0.01, **p = 0.04 by Welch’s t-test. **(B)** PD-1 expression is downregulated with rapamycin treatment (25 nM) during CD3/CD28 stimulation (72 h) in melanoma TILs. CD8^+^CD45RA-CCR7^−^CD27^−^ effector memory T cells are shown on the left (*p = 0.05, paired t-test), and CD8^+^CD45RA^−^ memory T cell trends are shown on the right (n.s.). **(C)** scRNAseq of CD8+ TILs in melanoma, visualized with UMAP dimensional reduction analysis, indicates nine unique clusters. **(D)** Pathway analysis of cluster 8 at single-cell resolution indicates 18 significantly enriched pathways (nominal p < 0.05) with positive enrichment of mTOR-related pathways, such as mTORC1 and PI3K-AKT-MTOR (shown in orange text). **(E)** Violin plots of expression of selected MTOR-related genes (PHGDH, E2F1, PSMA4, BUB1, and CDK2) in the scRNAseq clusters. mTOR, mammalian target of rapamycin; TILs, tumor-infiltrating lymphocytes; scRNAseq, single-cell RNA sequencing; UMAP, uniform manifold approximation and projection.

The mTORC1 signaling pathway was highly significantly enriched in tumors as compared to peripheral blood (**Figures 1C**, **3C**). Using intracellular flow cytometry, we conducted further analysis to discover that two of the key targets of mTORC1 complex activation, S6 ribosomal protein (target of S6 kinase, which is a direct target of mTOR phosphorylation) and 4EBP1 (a direct target of mTOR phosphorylation), were significantly more phosphorylated in the MEL TILs compared to peripheral CD8 T cells, validating our findings at the phosphoprotein level (**Figure 4A**). Furthermore, treatment with rapamycin (sirolimus), which specifically blocks MTORC1 signaling during TCR (anti-CD3/CD28) stimulation of melanoma TILs, resulted in consistent downregulation of PD-1 expression, particularly in the CD8^+^CD45RA^−^ memory T cells and CD8^+^CD45RA-CCR7^−^CD27^−^ effector memory T cells in the tumors (**Figure 4B**).

Moreover, to validate the differential enrichment of the mTOR pathway, we analyzed the single-cell transcriptomes of CD8^+^ TILs from a new cohort of metastatic melanoma patients collected prior to anti-PD-1 immunotherapy. Single-cell analysis of CD8 TILs also allowed us to compare distinct T cell populations within the tumor environment. UMAP dimensional reduction of the single-cell RNA sequencing (scRNAseq) dataset revealed a total of nine clusters (**Figure 4C**). We observed distinct clusters of cells with varying levels of T cell exhaustion. We observed high levels of T cell exhaustion markers PDCD1 (PD-1), HAVCR2 (TIM3), LAG3, CXCL13, and TOX in clusters 2, 4, and 8. Cells in these clusters expressed low levels of genes associated with non-exhausted T cells (TCF7 (TCF1), CCR7, SELL, and IL7R) (**Supplementary Figure 4A**). Cluster 8 demonstrated positive enrichment of the mTORC1 and PI3K/AKT/MTOR signaling pathways at the single-cell resolution, validating our bulkseq findings (**Figure 4D**). Violin plots of several genes associated with cluster 8 (PHGDH, E2F1, PSMA4, BUB1, CDK2, MKI67, IDH2, and FABP5) are shown in **Figure 4E** (mTOR related) and **Supplementary Figure 4B**, all of which were also differentially regulated in our bulk RNAseq signature of dysfunctional CD8^+^ TILs. We also confirmed PHGDH expression at the protein level (**Figure 3E**). Cluster 4 also displayed an exhausted T cell phenotype as well as enrichment of key metabolic pathways, including peroxisome, cholesterol homeostasis, and fatty acid metabolism (**Supplementary Figure 4C**). These results support our transcriptional analysis of flow-sorted MEL and SCC CD8^+^PD-1^+^TIM-3^+^ TILs and pCD8s (**Figure 1**) as well as our findings comparing MEL CD8+ TILs and pCD8s (**Figure 3**). Overall, our data suggest that CD8+ TIL exhaustion is associated with dysregulation of multiple pathways that warrant further investigation, including peroxisome, bile acid, and fatty acid metabolism, cholesterol homeostasis, and mTORC1 signaling.

## Discussion

Our highly focused transcriptomic profiling of dysfunctional CD8^+^PD-1^+^TIM-3^+^ TILs in human melanoma and SCC has identified several seemingly disparate and independent metabolic pathways that are differentially regulated in CD8^+^ Tex/act cells in the tumor microenvironment. Notably, we show that bile acid and peroxisome, along with mTOR pathways, are enriched in dysfunctional CD8^+^ Tex cells. Our findings reveal potential metabolic pathways that can be targeted to reinvigorate the dysfunctional immune system observed in melanoma and SCC-derived CD8^+^PD-1^+^TIM-3^+^ TILs.

Although reports have recently emerged showing the potential immunomodulatory effects of bile acids on the immune response, to the best of our knowledge, ours is the first report of bile acid metabolism pathway dysregulation in tumor-infiltrating lymphocytes in cancer (38–40). One of the key regulatory enzymes of the bile acid metabolism pathway is CYP27, a cytochrome P450 enzyme encoded by the CYP27A1 gene (41). CYP27 metabolizes cholesterol into 27-hydroxycholesterol (27HC) through a hydroxylation reaction. Current evidence indicates that 27HC can exert modulatory effects on the immune system and act as a selective estrogen receptor modulator (SERM) (42, 43). Strikingly, 27HC stimulates the proliferation of mouse melanoma cells by activating estrogen receptor alpha (ERα) and triggering the AKT and MAPK pathways (44). Compelling research into potential therapeutic strategies targeting bile acid metabolism can be found in breast cancer research (45, 46). For example, pharmacologic inhibition of CYP27A1 improves the efficacy of anti-PD-1 treatment and decreases metastatic breast cancer growth in mice (47).

The observation of dysregulated bile acid metabolism in melanoma and SCC TILs is also interesting given the considerable amount of data highlighting the relationship between antitumor immune response and the microbiome (48–50). The liver produces primary bile acids, which are then modified by gut microbes into a wide range of compounds with roles in gut metabolism, cell signaling, and microbe composition. Perturbations in the microbial ecosystem have been linked to immune evasion, carcinogenesis, and chronic inflammation and can affect the efficacy of cancer immunotherapies, including ICI (51). While most studies have focused on the gut microbiome and its connection to immune surveillance dysregulation, the interplay between cancer cells, immune cells, and the microbiota is also relevant in the tumor microenvironment (52, 53). Notably, it has been observed that numerous tumor types, including melanoma, have distinct microbial signatures. The abundance of specific intratumor bacteria in melanoma has been linked to CD8^+^ T cell infiltration and patient survival (54). Additionally, patient immunotherapy response has been linked to metabolic functions encoded by a unique pattern of intratumor bacteria (55). An intriguing possibility is that dysregulation of the bile acid metabolism pathway in CD8^+^ TILs is interconnected with tumor microbiome dysbiosis in the TME and may influence the anticancer immune response. It is plausible that manipulating the tumor-associated microbiome may also influence tumor immunity and patient response to immune therapy, as has been demonstrated in the case of the gut microbiome (48, 56, 57). Further investigation is needed to explore the interdependence of the tumor microbiome, tumor metabolic reprogramming, and CD8^+^ T cell dysfunction.

Upregulation of bile acid-related genes in tumor-infiltrating CD8^+^ T cells correlates with T cell dysfunction activity and may also be related to the differential regulation of metabolic-related pathways, such as mTORC1. Connections between bile acid and mTOR pathway activation have been reported. Yamada et al. showed that secondary bile acids activate the mTOR pathway via FXR signaling (58). The bile acid activation of mTOR signaling may be caused by the upregulation of DGKH (diacylglyerol kinase), an enzyme that converts diacylglycerol to phosphatidic acid, which directly activates mTOR (59). DGKH was significantly upregulated in tumor-infiltrating CD8^+^ T cells in melanoma and SCC in our study.

Our results are consistent with the hypothesis that transcriptional reprogramming is likely to play a role in driving the changes in cellular metabolism in dysfunctional CD8^+^ TILs in melanoma. TOX, a transcription factor that drives CD8 T cells toward an exhausted state, is upregulated in SCC and melanoma TILs when compared to pCD8s (**Figures 1B**, **3A**) (60), indicating that targeting TOX along with PD-1 could offset T cell exhaustion. Two key metabolic genes, PKM2 and PHGDH, are upregulated in melanoma TILs compared to healthy pCD8s (**Figures 1B**, **3A, E**). PKM2 (pyruvate kinase M1/2) expression, which controls glycolysis, has been found to be associated with tumor progression and poor survival rates in a small pilot study of metastatic melanoma patients (61). PHGDH (d-3-phosphoglycerate dehydrogenase) is overexpressed in multiple cancers, correlates with tumor growth, and has been shown to be increased in 40% of melanoma samples (62, 63). scRNAseq and UMAP analysis indicated that PHGDH, along with CDK2, E2F1, BUB1, and PSMA4, was associated with increased mTOR-related signaling in cluster 8 (**Figures 4C-E**). CDK2, E2F1, and BUB1 (mitotic checkpoint serine/threonine-protein kinase BUB1) are involved in cell cycle progression, and targeting them could prove to be a viable therapeutic strategy. Inhibition of CDK2 in melanoma cell lines has been shown to overcome their resistance to BRAF and Hsp90 inhibitors (64), while BUB1 has been identified as a novel target downstream of SIRT1 in melanoma (65). E2F1 is overexpressed in melanoma, and its inhibition initiates cell cycle arrest and apoptosis, as well as increasing the sensitivity of the melanoma cells to BRAF inhibitors (66). Finally, PSMA4 is upregulated in OT1 CD8^+^ T cells following transient and continuous Ag-independent stimulation (67).

Metabolic reprogramming is a hallmark of T cell exhaustion, driven by chronic antigen stimulation, hypoxia, and high levels of reactive oxygen species (68). As described in the Introduction, current models of T cell exhaustion have described two main subsets of exhausted T cells: “precursor exhausted” cells (Tpex, PD-1^+^, TCF1^+^, and TIM-3^−^) and “terminally exhausted” T cells (Tex, PD1^+^, TCF1^−^, and TIM-3^+^). Tpex cells mainly use mitochondrial fatty acid oxidation and oxidative phosphorylation (OXPHOS) for energy. In contrast, Tex cells rely more on glycolytic metabolism, as they have decreased mitochondrial membrane potential, which hinders their ability to utilize OXPHOS. Thus, T cell metabolic states are dynamic and change throughout the process of T cell exhaustion (69) (70) (71). Previously, we also showed that OXPHOS and glycolysis are upregulated in a subset of exhausted T cells (72). Consistent with these studies, our flow cytometric UMAP analysis (**Figure 2**) revealed clusters representative of different T cell populations with distinct metabolic characteristics. Dimension reduction of our multiparametric flow cytometry data from peripheral CD8 T cells and CD8^+^ TILs clearly demonstrated that the frequency of cells in clusters that expressed the highest levels of coordinate immune checkpoint receptor expression was significantly positively correlated with the enrichment of key metabolic pathways, including glycolysis, OXPHOS, peroxisome, fatty acid metabolism, and bile acid metabolism. We propose that TIL clusters 15, 16, and 6 are enriched for CD8^+^ Tex/act cells and may identify progressive levels of immune exhaustion/dysfunction in the CD8 compartment, with coordinate expression of ICR to maximal levels in cluster 6, whose frequency has significant correlation with enrichment of the bile acid metabolism pathway. These results highlight the dynamic nature of T cell differentiation from progenitor to terminal exhaustion, an important consideration in the development of strategies to intervene in and reverse certain stages of exhaustion in T cells.

These data were further supported by our scRNAseq analysis of melanoma CD8 TILs, in which we observed defined clusters of T cells associated with varying levels of T cell exhaustion markers. Notably, clusters 4 and 8 expressed high levels of the exhaustion markers (PDCD1, HAVCR2, LAG3, CXCL13, and TOX) and were associated with the upregulation of many of the metabolic pathways identified in our other analyses, including mTORC1 signaling (cluster 8) and peroxisome, fatty acid metabolism, and cholesterol homeostasis (cluster 4).

In the subset of our patients who received anti-PD-1 immunotherapy (**Figure 3F**), we observed upregulation of CXCR6 and CTTNBP2NL and downregulation of CD160 and HRH2. CXCR6, C-X-C chemokine receptor type 6, is known to be preferentially expressed on CD8^+^PD-1^high^ exhausted T cells in hepatocellular carcinoma (HCC) and is hypothesized to play a role in recruiting CD8^+^ T cells into the tumor microenvironment (73). Overexpression of CTTNBP2NL and subsequent STRIPAK complexes could fuel the exhausted CD8^+^ T cells, thus suppressing immunotherapy effects and promoting tumor progression. These overexpressed refractory gene profiles open new avenues for novel immunotherapy targets. CD160, a degranulation marker, was downregulated in our immune failure signature. Its expression has been demonstrated to be downregulated with repeated antigen stimulation, while PD-1 and LAG3 remained high and TIM-3 increased further in T cells from a murine breast cancer model (74). Furthermore, in CD8^+^ T cells from individuals with HIV, CD160 and PD-1 co-expression represent a subset of exhausted T cells both functionally and transcriptionally (75). Therefore, downregulation of CD160 may indicate dysfunctional TILs that either are no longer degranulating or lack continued TCR engagement. Finally, downregulation of HRH2 (histamine receptor H2) could be indicative of tumor metastasis in the context of immune failure, as a balance of histamine with its receptor is necessary to either stimulate or suppress the growth of melanoma (76). Downregulation of HRH2 and CD160 was also observed in the combined immunotherapy signature (**Supplementary Figure 3**), along with downregulation of the fatty acid metabolism, glycolysis, and mTORC1 signaling pathways. Our data suggest that these pathways are commonly upregulated in exhausted T cells; therefore, they may represent unique therapeutic targets.

While we did not investigate whether altered metabolic dysregulation can be predictive of anti-PD-1 immunotherapy response, there is evidence that metabolic signaling pathways can serve as candidate biomarkers for ICI therapy efficacy, notably from a study linking increased oxidative metabolism in tumors to decreased likelihood of response to anti-PD-1 immunotherapy in melanoma patients (77). Melanoma patients who responded to anti-PD-1 exhibited increased glycolysis, fatty acid metabolism, and tryptophan and branched-chain amino acid metabolism in their PBMCs, indicative of enhanced mitochondrial function during periods of stress. In the same patients, CD8^+^ cells had high levels of SLC2A14 and LDHC (78). Additionally, urological cancer patients who responded to nivolumab had higher levels of long-chain fatty acids in serum compared to non-responders (79). Our results indicate that dysregulation of the peroxisome and bile acid metabolism and mTOR signaling pathways may also serve as candidate predictors of immunotherapy response.

Our current systems biology analysis of human melanoma was conducted in an unbiased fashion to fully interrogate dysfunctional CD8^+^PD-1^+^TIM-3^+^ TIL (sorted) transcriptomic profiles, allowing for the identification of novel and unexpected signaling pathways that may reveal enhancement targets in more recent immune checkpoint blockade therapies. Taken together, our findings indicate that mTOR, bile acid metabolism, and peroxisome metabolic pathways are coordinately dysregulated and highly upregulated in the CD8^+^ TILs of melanoma and SCC patients, impacting the capacity of the host immune system to suppress and eradicate tumor cells. Unraveling of the complex metabolic reprogramming that occurs during T cell exhaustion will pave the way for tailored therapeutic approaches that effectively target unique metabolic subsets of exhausted T cells. This has the potential to reinvigorate resistance to immune checkpoint therapy failure, thus improving clinical efficacy. Our results provide a new platform and public resource for data mining and for the development of novel treatment modalities to further improve current immunotherapy advancement.

## Methods

### Patient selection and demographics

Samples were obtained at the Cleveland Clinic under a protocol approved by the Cleveland Clinic’s institutional review board, and written informed consent was obtained from each patient. Peripheral blood lymphocytes (PBLs) and tumor specimens were obtained from patients with cutaneous melanoma (MEL, n = 23), SCC (n = 8), and control individuals without skin disease (n = 8), as previously described (29). Of the MEL patients, 60.9% were male, with a mean age of 58.3 ( ± 15.8); three patients presented with a primary tumor, while 20 patients had metastatic disease. Fourteen of the MEL patients received some type of immunotherapy (IO); out of these patients, eight received anti-PD-1/PD-L1 directed IO (either pembrolizumab or nivolumab). Of the SCC patients, 85.7% were male, with a mean age of 73 ( ± 7.9); three patients presented with a primary tumor, while five patients had metastatic disease. Age was unknown for one SCC patient. Tissue for scRNAseq libraries was obtained from patients with cutaneous melanoma (n = 8), of whom 37.5% were male, with a mean age of 60 ( ± 12.7). Two patients presented with a primary tumor, while six patients had metastatic disease. All tumor specimens came from different individuals. Clinical data are summarized in **Supplementary Table 1**.

### Isolation of TILs and PBLs

After surgical resection, tumor specimens were washed with antibiotic-containing media and minced with crossed scalpels under sterile conditions. Tissue was dissociated via enzymatic digestion using 1,500 U/ml collagenase IV (Gibco, Grand Island, NY, USA/Life Technologies, Carlsbad, CA, USA), 1,000 U/ml hyaluronidase (Sigma, St. Louis, MO, USA), and 0.05 mU/ml DNase IV (Gibco) in Roswell Park Memorial Institute (RPMI) medium for 1 h at 37°C followed by mechanical agitation. Centrifugation over a Ficoll–Hypaque gradient was used to separate debris from the single-cell suspension. Finally, cells were cryopreserved in 10% dimethyl sulfoxide (DMSO) + bovine serum. Similarly, peripheral blood mononuclear cells were purified from buffy coats by centrifugation over a Ficoll–Hypaque gradient, followed by cryopreservation.

### Flow cytometric analysis

Flow cytometric analysis of immune checkpoint receptor and activation marker expression and T cell memory subset distribution was carried out as follows. For memory subset phenotyping and assessment of negative regulator and ligand expression, a cocktail of the following monoclonal antibodies was used: anti-CD3 Alexa700 (BD, San Jose, CA, USA), anti-CD4 Q.605 (Invitrogen, Carlsbad, CA, USA), anti-CD8 PerCP (BioLegend, San Diego, CA, USA), anti-CD45RA BV650 (BioLegend), anti-CD27 APC-eFluor 780 (eBioscience, San Diego, CA, USA), anti-CCR7 PE-CF594 (BD), anti-CD14 V500 (BD), anti-CD19 BV510 (BioLegend), Live/Dead Amcyan (Invitrogen), anti-BTLA (BD), anti-TIM3 BV421 (BioLegend), anti-PD-1 PE-Cy7 (BioLegend), anti-CTLA-4 APC (BD), anti-TIGIT PE (eBioscience), and anti-LAG3 FITC (Novus, Centennial, CO, USA). After washing, cells were resuspended in staining buffer and sorted on an ARIA-SORP. The following antibodies were used for intracellular flow cytometry to assess the phosphorylation of mTOR targets: anti-S6 (S235/S236) V450 (BD) and anti-p4E-BP1 (T36/46) PE (BD). For the rapamycin assay, cells were stimulated for 3 days with CD3/CD28 beads and then treated with 25 nM of rapamycin overnight. Cells were stained and analyzed for PD-1 surface expression. The following antibodies were used to identify memory T cells and effector memory T cells: anti-CD8 BV711 (BD), anti-CD45RA BV650 (BioLegend), anti-CCR7 FITC (R&D Systems, Minneapolis, MN, USA), and anti-CD27 APC eFluor 780 (eBioscience). Data were analyzed using FlowJo software (TreeStar) and our custom pipeline for dimensionality reduction.

### Western blotting

Isolated CD8 cells (50,000) from TILs were lysed in 50 μl of radioimmunoprecipitation assay (RIPA) buffer (150 mM of NaCl, 1 mM of EDTA, 0.5% Triton X-100, 0.5% deoxycholic acid, 0.5% sodium dodecyl sulfate (SDS), and 100 mM of Tris (pH 7.5) containing protease and phosphatase inhibitors). An equal sample volume (100 μl) was used for reducing gel electrophoresis (4%–12%), followed by immunodetection with antibodies toward CYP27A1 (Abcam, Cambridge, UK), Actin (Abcam), PHGDH (Cell Signaling, Danvers, MA, USA), PKM2 (Cell Signaling), and PCK2 (Cell Signaling). Three biological replicates were performed.

### Bulk RNAseq and bioinformatic analysis

RNA was purified from CD8 T cells using RNeasy Micro Kits (Qiagen, Hilden, Germany), followed by low-input RNASeq library generation using Takara SMART-Seq v4 Ultra Low/Nextera XT with Nextera Index v2 Set A. Paired-end sequencing reactions were run on an Illumina NextSeq 550 High Output platform (25M total reads per sample). Raw demultiplexed fastq paired-end read files were trimmed of adapters and filtered using the program skewer to remove reads with an average Phred quality score of less than 30, or trimmed to a length of less than 36 (80). Trimmed reads were then aligned using the HISAT2 aligner to the Homo sapiens NCBI reference genome assembly version GRCh38 and sorted using SAMtools (81, 82). Aligned reads were counted and assigned to gene meta-features using the program featureCounts as part of the Subread package (83). These count files were imported into the R programming environment and were assessed for quality control, normalized, and analyzed via the limma-trend method (84) for differential gene expression testing as well as regression modeling and GSVA (85). Statistical power was guided by the detection of a twofold gene expression change in ≥8 samples per group at a minimum power of 80% at p = 0.05, assuming a sequencing depth between 5× and 8× and a coefficient of variation of 0.4 (commonly used for human studies) (86). Linear regression modeling was performed using the limma framework. RNAseq data supporting this study have been deposited in the Gene Expression Omnibus (GEO) public database with the accession number pending.

### scRNAseq library preparation and data processing

All cells were resuspended in Dulbecco’s phosphate-buffered saline (DPBS) with 0.04% bovine serum albumin (BSA) and immediately processed for scRNAseq, as follows. Cell count and viability were determined using trypan blue on a Countess FL II, and approximately 12,000 cells were loaded for capture onto the Chromium System using the v2 single-cell reagent kit according to the manufacturer’s protocol (10X Genomics, Pleasanton, CA, USA). Following capture and lysis, cDNA was synthesized and amplified (12 cycles) as per the manufacturer’s protocol (10X Genomics). The amplified cDNA from each channel of the Chromium System was used to construct an Illumina sequencing library and was sequenced on an Illumina HiSeq 2500 with 150-cycle sequencing (asymmetric reads per 10X Genomics). Illumina basecall files (*.bcl) were converted to FASTQs using CellRanger v3.0, which uses bcl2fastq v2.17.1.14. FASTQ files were then aligned to the GRCh38 human reference genome and transcriptome using the CellRanger v3.0 software pipeline with default parameters, as reported previously (87); this demultiplexes the samples, generates a gene-versus-cell expression matrix based on the barcodes, and assigns UMIs that enable determination of the individual cell from which the RNA molecule originated. Overall, an estimated total of 71,238 cells were analyzed.

### scRNAseq bioinformatic analysis

Bioinformatic analysis of cells based on whole transcriptomes was performed using the R package Seurat (version 3.0) (88). The dimensionality of gene–barcode matrices was first reduced to 18 principal components using principal component analysis (PCA). PCA-reduced data were further reduced to two-dimensional space using the UMAP method and visualized. Graph-based clustering of cells was conducted in the PCA space; a sparse nearest-neighbor graph of the cells was built first, and Louvain modularity optimization was then applied. The number of nearest neighbors was logarithmic in accordance with the number of cells. In the last step, repeated cycles of hierarchical clustering and merging of cluster pairs that had no significant differential expression was performed, until no more cluster pairs could merge. Differential gene expression analyses of each cluster were conducted using the Wilcoxon rank sum test. The log2 fold-change in expression of a certain gene (UMIs) in one cluster *vs.* all other clusters, and the corresponding adjusted p-values, were calculated for each cluster, with pathway enrichment scores generated using the GSEA method (89).

### Statistics

Unless otherwise indicated, the Student’s t-test was used, with p ≤ 0.05 chosen as the level of significance, and analyses were performed using GraphPad Prism 8.

## Data availability statement

The bulk and single cell RNA sequencing datasets have been deposited to the NCBI Gene Expression Omnibus with the accession numbers GSE230864 and GSE138720, respectively.

## Ethics statement

The studies involving humans were approved by Cleveland Clinic’s Institutional Review Board. The studies were conducted in accordance with the local legislation and institutional requirements. The participants provided their written informed consent to participate in this study.

## Author contributions

BG, YP, LP, ST, and GR conducted the clinical work, collected the samples, and analyzed the clinical data. CC, BR, JBG, BT, X-HG, LZ, and MC conducted the immune and transcriptomic assays and bioinformatic analysis. BG designed and oversaw the clinical studies. BG and MC secured funding for the study. CC, BR, JBG, YP, LZ, MC, and BG wrote the manuscript. All authors contributed to the article and approved the submitted version.

## Supplementary material

Supplementary Figure 1 Characterization of dysfunctional CD8+ T cells in total TILs and PBMC. **(A)** Venn analysis identified genes that are common and unique in total (melanoma and SCC) CD8^+^PD-1^+^TIM-3^+^ TILs (nominal p ≤ 0.05). 2,057 genes were commonly differentially regulated in both total CD8^+/^P^+/^T^+^ TIL and peripheral CD8^+^ T cells, while 2,223 and 789 were unique to melanoma and SCC CD8^+/^P^+/^T^+^ TILs, respectively, in comparison to healthy peripheral CD8^+^ T cells. **(B)** Core biological pathways associated with TGF-β attenuation of the tumor response to immune checkpoint blockade were significantly differentially regulated within the dysfunctional CD8^+/^P^+/^T^+^ melanoma and SCC TILs, as shown by the two-way heatmap. Pathways and genes (noted on the right y-axis) include those involved in FGFR3 signaling (TP63), CD8 T cell effector function (GZMA, GZMB, PRF1 and CXCL10), immune checkpoint receptors and their ligands (PDCD1LG2, CTLA4, PD-1, LAG3, TIM-3, and TIGIT), cell cycle (MKI67, CCNE1, BUB1, BUB1B, CCNB2, CDC25C, CDK2, MCM4, and MCM6), DNA damage response (BRCA2, ERCC2, FANCA, and RAD51c), and TGF-β response signature genes (ACTA2 and COL4A1). HLA-B and TGFB1 genes were uniquely downregulated in the CD8^+^ TIL as compared to PBMC. All p ≤ 0.05.

Supplementary Figure 2 Hallmark pathway enrichment in melanoma TILs. Top 20 differentially enriched pathways between melanoma TILs and pCD8s with associated top 50 shared differentially expressed genes. Pathways and genes were selected based on a nominal p ≤ 0.05 and the number of genes in common between pathways. Heatmap values show the logFC values for each gene between melanoma TILs and pCD8s, while their column annotations denote p values. Row annotations denote the logFC values of pathway enrichment scores between the two groups.

Supplementary Figure 3 Identification of transcriptomic signatures common to broad immunotherapy treatments. **(A)** Top 50 differentially expressed genes p ≤ 0.05 by logFC between CD8 TIL immunotherapy and no immunotherapy in melanoma and SCC patients. Immunotherapy type, clinical outcome, and cancer type are denoted in the legend on the left. Immunotherapy – NO: no immunotherapy, IPI.PEMBRO: ipilimumab + pembrolizumab, IPI.NIVO: ipilimumab + nivolumab, IPI: ipilimumab, IO: immunotherapy, NIVO: nivolumab, PEMBRO: pembrolizumab. Outcome – NED: no evidence of disease. Cancer type – SCC: squamous cell carcinoma, CUT MEL: cutaneous melanoma, MUC MEL: mucosal melanoma. **(B)** Hallmark pathways downregulated in CD8 TILs in patients treated with immunotherapy.

Supplementary Figure 4 Characterization of CD8 TILs at the single-cell level. **(A)** Log2 sum expression of selected genes is shown across UMAP clusters from . PDCD1, HAVCR2, LAG3, CXCL13, and TOX were used as markers of exhausted CD8 T cells. TCF1, CCR7, SELL and IL7R were used as markers of non-exhausted CD8 T cells. **(B)** Expression pattern of individual genes of interest (MKI67, IDH2, FABP5) across UMAP clusters. **(C)** KEGG and Hallmark pathways enriched (nominal <0.05) in cluster 4. Pathways of interest are highlighted in red. NES: normalized enrichment score.

Supplementary Table 1 Clinical information associated with tumor specimens. Information on tumor cancer type (cutaneous or mucosal melanoma, primary or metastatic), immunotherapy treatment, and patient outcomes.
