# Supplementary figures and images for: A transcriptional evaluation of the melanoma and squamous cell carcinoma TIL compartment reveals an unexpected spectrum of exhausted and functional T cells
Source: Front Oncol. 2023 Oct 30;13:1200387. doi: 10.3389/fonc.2023.1200387 (PMC10643547; doi:10.3389/fonc.2023.1200387)

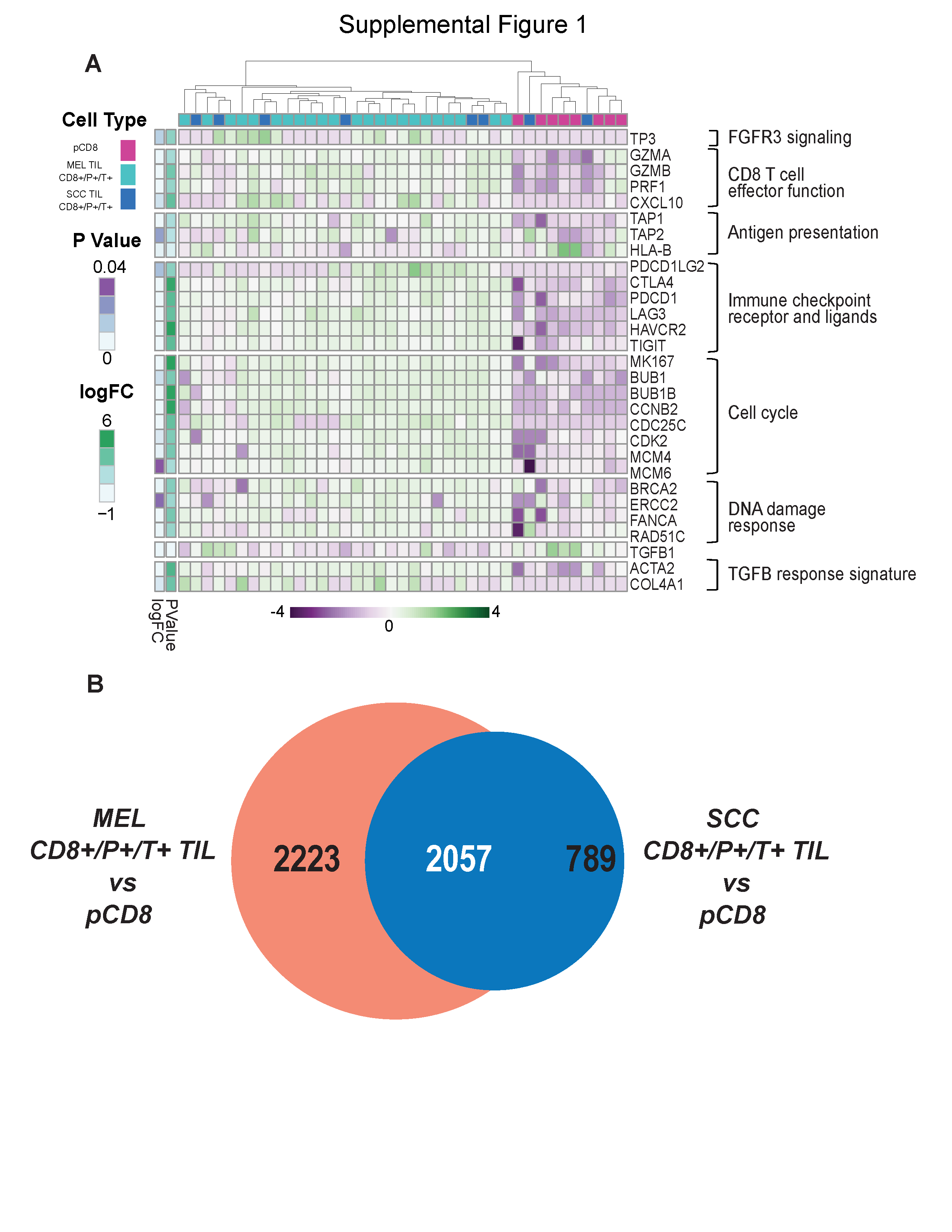

Supplement: Supplementary Figure 1 — Characterization of dysfunctional CD8+ T cells in total TILs and PBMC. (A) Venn analysis identified genes that are common and unique in total (melanoma and SCC) CD8+PD-1+TIM-3+ TILs (nominal p ≤ 0.05). 2,057 genes were commonly differentially regulated in both total CD8+/P+/T+ TIL and peripheral CD8+ T cells, while 2,223 and 789 were unique to melanoma and SCC CD8+/P+/T+ TILs, respectively, in comparison to healthy peripheral CD8+ T cells. (B) Core biological pathways associated with TGF-β attenuation of the tumor response to immune checkpoint blockade were significantly differentially regulated within the dysfunctional CD8+/P+/T+ melanoma and SCC TILs, as shown by the two-way heatmap. Pathways and genes (noted on the right y-axis) include those involved in FGFR3 signaling (TP63), CD8 T cell effector function (GZMA, GZMB, PRF1 and CXCL10), immune checkpoint receptors and their ligands (PDCD1LG2, CTLA4, PD-1, LAG3, TIM-3, and TIGIT), cell cycle (MKI67, CCNE1, BUB1, BUB1B, CCNB2, CDC25C, CDK2, MCM4, and MCM6), DNA damage response (BRCA2, ERCC2, FANCA, and RAD51c), and TGF-β response signature genes (ACTA2 and COL4A1). HLA-B and TGFB1 genes were uniquely downregulated in the CD8+ TIL as compared to PBMC. All p ≤ 0.05. [file Image_1.tiff]

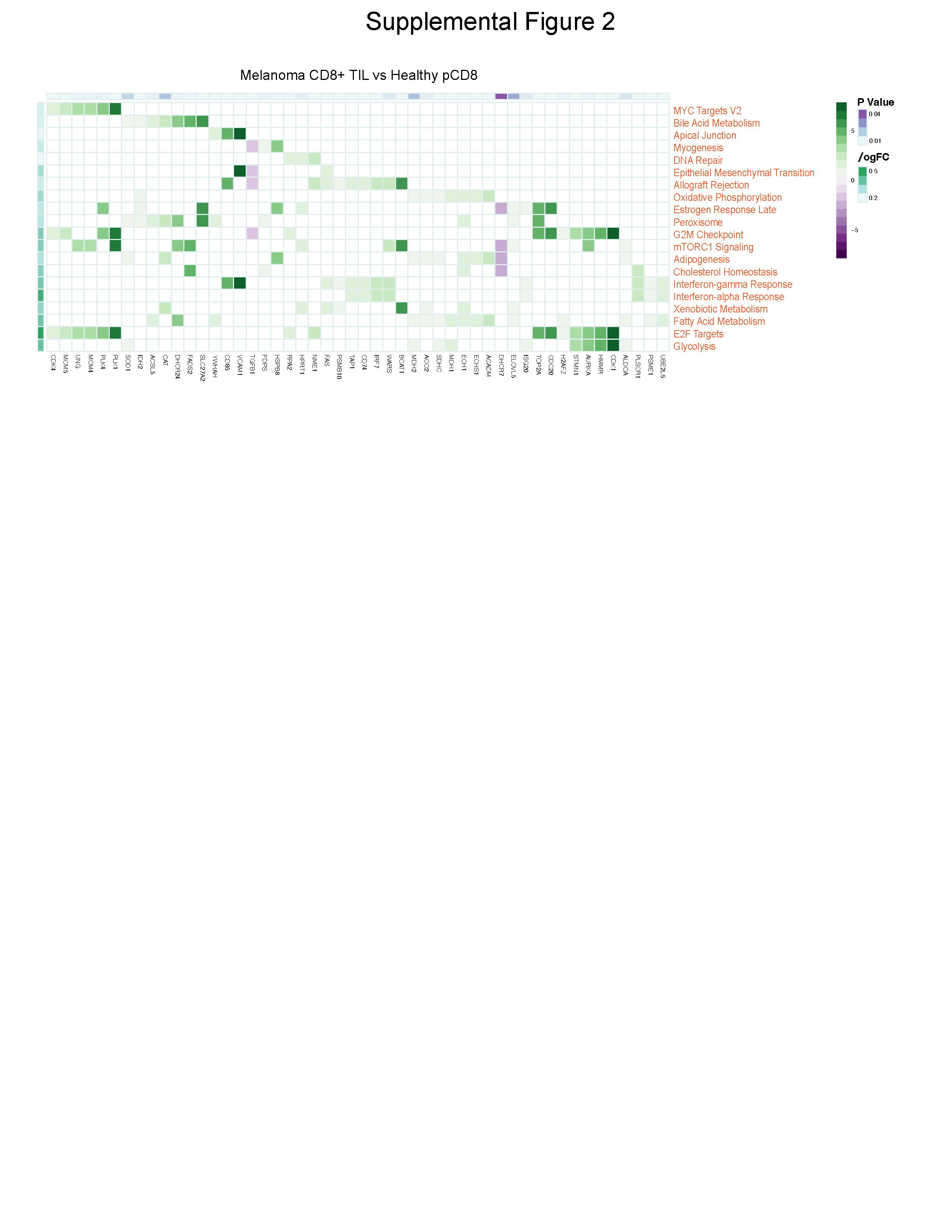

Supplement: Supplementary Figure 2 — Hallmark pathway enrichment in melanoma TILs. Top 20 differentially enriched pathways between melanoma TILs and pCD8s with associated top 50 shared differentially expressed genes. Pathways and genes were selected based on a nominal p ≤ 0.05 and the number of genes in common between pathways. Heatmap values show the logFC values for each gene between melanoma TILs and pCD8s, while their column annotations denote p values. Row annotations denote the logFC values of pathway enrichment scores between the two groups. [file Image_2.tiff]

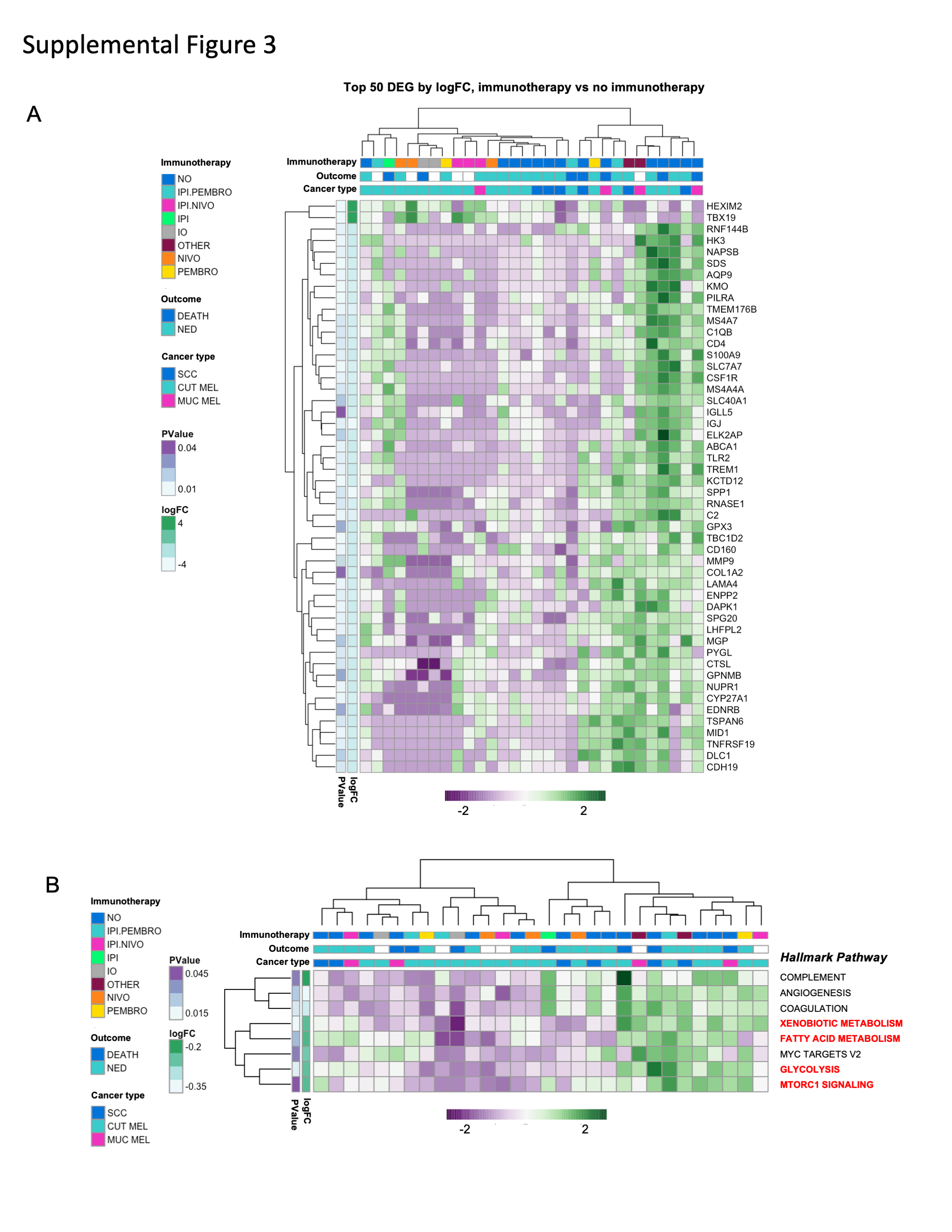

Supplement: Supplementary Figure 3 — Identification of transcriptomic signatures common to broad immunotherapy treatments. (A) Top 50 differentially expressed genes p ≤ 0.05 by logFC between CD8 TIL immunotherapy and no immunotherapy in melanoma and SCC patients. Immunotherapy type, clinical outcome, and cancer type are denoted in the legend on the left. Immunotherapy – NO: no immunotherapy, IPI.PEMBRO: ipilimumab + pembrolizumab, IPI.NIVO: ipilimumab + nivolumab, IPI: ipilimumab, IO: immunotherapy, NIVO: nivolumab, PEMBRO: pembrolizumab. Outcome – NED: no evidence of disease. Cancer type – SCC: squamous cell carcinoma, CUT MEL: cutaneous melanoma, MUC MEL: mucosal melanoma. (B) Hallmark pathways downregulated in CD8 TILs in patients treated with immunotherapy. [file Image_3.tiff]

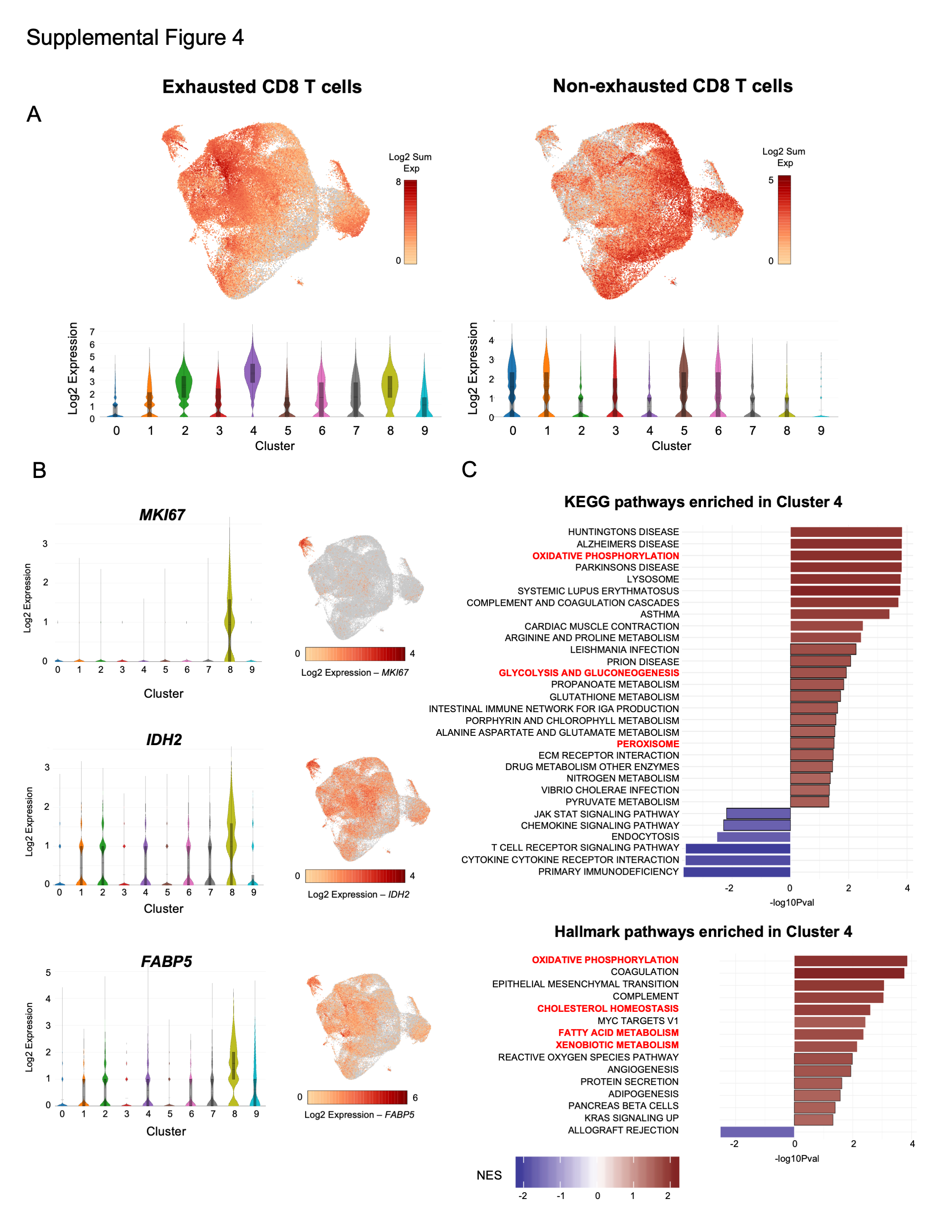

Supplement: Supplementary Figure 4 — Characterization of CD8 TILs at the single-cell level. (A) Log2 sum expression of selected genes is shown across UMAP clusters from . PDCD1, HAVCR2, LAG3, CXCL13, and TOX were used as markers of exhausted CD8 T cells. TCF1, CCR7, SELL and IL7R were used as markers of non-exhausted CD8 T cells. (B) Expression pattern of individual genes of interest (MKI67, IDH2, FABP5) across UMAP clusters. (C) KEGG and Hallmark pathways enriched (nominal <0.05) in cluster 4. Pathways of interest are highlighted in red. NES: normalized enrichment score. [file Image_4.tiff]
